# Development and characterization of gelatin-based biodegradable films incorporated with pistachio shell hemicellulose

**DOI:** 10.1007/s13197-024-05968-4

**Published:** 2024-03-17

**Authors:** Narjes Harrazi, Hatice Neval Özbek, Derya Koçak Yanık, Imen Zaghbib, Fahrettin Göğüş

**Affiliations:** 1 Higher School of Food Industries of Tunis, University of Carthage, 1003 Tunis, Tunisia; 2 Department of Food Engineering, Engineering Faculty, University of Gaziantep, 27310 Gaziantep, Turkey; 3 Department of Food Engineering, Faculty of Agriculture, Eskişehir Osmangazi University, 26040 Eskisehir, Turkey; 4 Higher Institute of Biotechnology of Béja, University of Jendouba, 9000 Beja, Tunisia

**Keywords:** Pistachio shell, Hemicellulose, Gelatin, Biodegradable film

## Abstract

This study aimed to incorporate pistachio shell hemicellulose into a film of gelatin and glycerol for the production of biodegradable films. The gelatin and glycerol are chosen because of their functional properties, which make it extensively used in food industry. The film composition was defined after a statistical optimization by central composite face-centered design and response surface methodology. The hemicellulose/gelatin ratio of 35.93% and the glycerol ratio of 18.02% were the optimum conditions to obtain lower film water solubility, higher tensile strength, and elongation at break values. The physical, structural, mechanical, and barrier properties of the developed hemicellulose-gelatin film were analyzed and compared with those of the gelatin film. Tensile strength and film water solubility values were reduced significantly with hemicellulose incorporation from 20.41 to 16.64 MPa and 49.57 to 39.21%, respectively, while EB was enhanced by 4.34 times. In addition, hemicellulose incorporation enhanced the water vapor permeability and the film degradation in the soil. The films were also examined by Fourier transform infrared spectroscopy and differential scanning calorimetry. The novelty of this study is to use pistcahio shell hemicellulose in the production of an edible film for the first time.

**Supplementary Information:**

The online version contains supplementary material available at 10.1007/s13197-024-05968-4.

## Introduction

Plastic is the most common industrial packaging all over the world. Plastics used in different proportions are: low-density polyethylene (LDPE) made up 31% of the plastic resins, polyethylene terephthalate (PET) 23%, polyvinyl chloride (PVC) 15%, polypropylene (PP) 12%, polystyrene (PS) 10% and high-density polyethylene (HDPE) 9%, due to their great packaging properties such as good oil and chemical resistance, excellent gas and water vapor barrier properties, high processability, and thermal stability (PAGEV 2019; Kim et al. 2014). However, these materials are non-renewable and also practically non-biodegradable in the environment, which causes environmental problems.

Recently, research has attempted to replace these materials with bio-based and biodegradable materials. Scientists concentrate on finding alternative and sustainable sources to produce eco-friendly biopolymers with good plastic properties based on renewable and natural raw materials (Wu et al. 2021). Polysaccharides such as cellulose, hemicellulose, and lignin are the main subjects of low-cost bio-sourced products. After cellulose, hemicellulose is arguably the second most abundant carbohydrate (Zhao et al. 2020). According to Christopher (2012) this polymer is valuable for several applications owing to its ability to form edible, biodegradable, and eco-friendly films. Hemicellulose is considered as the cell wall polysaccharide, exempting cellulose and pectin. Contrary to cellulose, hemicelluloses are branched and have attached side groups. They are qualified as short heteropolymers composed of xyloglucans, xylans, mannans, glucomannans, and β-(1 → 3,1 → 4)-glucans (Scheller and Ulvskov 2010). Hemicellulose is used to produce films because of its film-forming properties, their biocompatibility, and biodegradability. Furthermore, hemicellulose-based films are known to exhibit gas barrier properties, especially toward oxygen (Chadni et al. 2020).

The pistachio is the most consumed tree nut in Turkey and is widely cultivated. Turkey is also one of the leading producers of pistachio nuts, with an annual production of 210,000 tons (in-shell bases) in 2022/23 (USDA 2023). Pistachio shell, which is an agro-waste, was qualified as one of the best hemicellulose sources because of its renewability, biodegradability, and non-toxicity (Sutivisedsak et al. 2012; Özbek et al. 2020). The incorporation of plasticizers such as glycerol, sorbitol, and xylitol helped to produce continuous and self-supporting hemicellulose films (Chadni et al. 2020). A co-matrix agent is added mostly to improve the film’s formability (Shao et al. 2019). The gelatin is known for its excellent functional properties that make it extensively used in the food industry, especially in desserts, candies, bakery products, jelly meat, ice cream, and dairy products. According to Luo et al. (2022), gelatin-based films provide good mechanical properties, while the incorporation of a plasticizer improves their functional activities.

This study is realized with the aim of evaluating the effect of the incorporation of hemicellulose, extracted from pistachio shell, on the gelatin-glycerol film as well as characterizing and defining hemicellulose-gelatin film properties. The glycerol was used as a plasticizer while the gelatin was used to improve film structure. The optimal film composition with good tensile strength (TS) and elongation properties was defined by varying the glycerol, the gelatin, and the hemicellulose content. The percentages of each substance were determined by using design expert. There are only a few studies using hemicellulose in edible film packaging composition, and as far as we are concerned, this is the first study including pistachio shell hemicellulose in film production.

## Materials and methods

### Materials

Pistachio shells were obtained from Altin Fistik Gida Mad. San. Ve Tic. Ltd. Şti., Gaziantep, Turkey. The shells were crushed to obtain a powder whose granule diameter does not exceed 425 µm. The obtained powder was kept at room temperature in a sealed bag. Sodium hydroxide, ethanol, glycerol, hydrochloric acid, and other chemicals were procured from Sigma Aldrich (St. Louis, MO, USA). Gelatin (cattle) was supplied from Sel Sanayi Ürünleri Ticaret ve Pazarlama A.Ş., Istanbul, Turkey.

### Hemicellulose extraction from pistachio shells

Hemicellulose was extracted from pistachio shells using the alkaline extraction method, followed by alcohol precipitation according to the procedure illustrated by Shao et al. (2019), with some modifications. The pistachio shell powder was mixed with a 9.5% (w/v) NaOH solution with a solid to liquid ratio of 1:20 (w/v). The obtained mixture was stirred for 4.2 h at 78 °C to solubilize hemicellulose. At the end of the time, the pH of the solution was adjusted to 5.5 using hydrochloric acid (37%). The mixture was subjected to centrifugation (5810 R, Eppendorf, Germany) at 10 000 rpm and 25 °C for 15 min for the separation of solid and liquid fractions. Then, ethanol (95%, 1:3 v/v) was added to the solution to precipitate hemicellulose, and the solution was stored overnight at 5 °C. At the end, the hemicellulose precipitate was dried for 48 h by a freeze dryer (Alpha 1–4 LD plus, Christ, Germany) to yield hemicellulose powder.

### Preparation of hemicellulose-gelatin films

The films were produced as stated by the experimental design with two variables: the hemicellulose/gelatin ratio (20–80 wt%) and glycerol ratio (15–45 wt% of hemicellulose and gelatin). Briefly, a weighed amount of the hemicellulose was dissolved in 30 mL of distilled water using a homogenizer (T18 Digital Ultra-Turrax, IKA, Germany) at 10,000 rpm for 15 min. The gelatin was also dissolved in water (30 mL) with stirring and heating at 80 °C. Then, the weighed amounts of glycerol were added to the gelatin solution and agitated again for 5 min. Then, the gelatin-glycerol mixture was added to the aqueous solution of hemicellulose, the final volume was set to 60 mL, and the whole solution was mixed for 5 min at 10,000 rpm. The concentration of the final solution was 5 g/100 mL. The well-proportioned solutions were treated in an ultrasonic bath (Sonorex Digiplus DL 255 H, Bandelin, Germany) at 35 kHz for 15 min, poured into 9 cm plastic petri dishes (10 mg/cm^2^), and dried at ambient conditions for 24 h. The gelatin film (gelatin + glycerol) was used as the control sample for all the analytical parameters. The films were kept at 25 °C and 50 ± 2% relative humidity in a closed chamber comprising a saturated magnesium nitrate solution before the analysis.

### Design of experiments and optimization of film production

A two-factor, three-level central composite face-centered design (CCFD) was used to evaluate the factors affecting the film properties. The independent variables were the hemicellulose/gelatin ratio (X_1_, 20–80 wt%) and the glycerol ratio (X_2_, 15–45 wt% of hemicellulose and gelatin). The responses were film water solubility (FWS), tensile strength (TS), and elongation at break (EB). 13 experiments containing 5 replicates at the central point were carried out in random order (Table 1). The statistical software of Design Expert v. 7.0 (Stat-Ease, Inc., Minneapolis, MN, USA) was used to design experiments and make statistical analyses with a significance level of 95%. The analysis of variance (ANOVA) was applied to estimate the significance of the independent factors and their interactions, the adequacy of the developed model, and the statistical significance of the regression coefficients.

**Table 1:** Central composite face-centered design generated for film production with the responses

Run no	X_1_ (%)	X_2_ (%)	FWS (%)	TS (MPa)	EB (%)
1	20	15	32.83 ± 1.35	22.99 ± 1.73	1.02 ± 0.07
2	80	15	46.14 ± 1.48	6.92 ± 0.28	4.50 ± 0.09
3	20	45	51.39 ± 1.86	12.26 ± 0.59	35.62 ± 1.05
4	80	45	53.32 ± 1.71	2.25 ± 0.08	14.60 ± 0.73
5	20	30	41.85 ± 1.36	22.15 ± 1.41	9.02 ± 0.21
6	80	30	49.02 ± 2.14	6.02 ± 0.34	8.75 ± 0.13
7	50	15	41.19 ± 1.97	11.29 ± 0.87	22.49 ± 1.09
8	50	45	50.53 ± 1.91	1.79 ± 0.06	55.83 ± 2.12
9	50	30	45.89 ± 1.24	10.52 ± 0.94	34.16 ± 1.09
10	50	30	46.29 ± 1.09	9.10 ± 0.71	40.34 ± 2.07
11	50	30	46.15 ± 1.34	9.25 ± 0.67	38.72 ± 1.69
12	50	30	45.65 ± 1.71	8.95 ± 0.72	35.6 ± 1.81
13	50	30	47.00 ± 2.16	9.16 ± 0.62	36.8 ± 1.57

*X*_*1*_ Hemicellulose/gelatin ratio (%), *X*_*2*_ Glycerol ratio (%), *FWS* Film water solubility (%), *TS* Tensile strength (MPa), *EB* Elongation at break (%)

The optimum conditions for film formulation were defined by the optimization function of the software, that resulting in minimum FWS, maximum TS, and EB. The optimum conditions were verified experimentally by preparing films in three replications.

### Analytical methods

#### Film thickness and moisture content

The thickness of the samples was determined by a digital caliper (Mitutoyo, Japan). Five measurements were taken between the edge and the center of each film. Results are averaged and expressed in mm with 0.001 mm accuracy. The moisture content of the films was defined by drying the samples (2 cm × 2 cm) in an oven for 24 h at 105 °C.

#### Film water solubility (FWS)

Using the method described by Kocabaş et al. (2021), the obtained films were cut in 3 cm × 3 cm dimensions and dried at 70 °C until constant weight (m_1_). Then, the samples were submerged in 10 mL of water and kept in a shaker incubator (Incubator 1000, Heidolph, Germany) for 1 h at 150 rpm. After incubation, the solution was filtrated, the solid particles were separated and dried again at 70 °C overnight (m_2_). The FWS was determined using Eq. 1.

1 $$FWS\left(\%\right)=\frac{({m}_{1}-{m}_{2})}{{m}_{1}}\times 100$$

#### Color

A colorimeter (model A-60-1010–615, HunterLab color Flex, USA) was used to evaluate the film color. The lightness (L*), yellowness (b*), and redness (a*) values of the films were determined. The total color difference (ΔE*), whiteness index (WI), and yellowness index (YI) were determined according to Eqs. 2, 3 and 4.

2 $$\Delta {E}^{*}=\sqrt{{(\Delta {L}^{*})}^{2}+{(\Delta {a}^{*})}^{2}+{(\Delta {b}^{*})}^{2}}$$

3 $${\text{WI}}=100-\sqrt{{(100-{L}^{*})}^{2}+{({a}^{*})}^{2}+{({b}^{*})}^{2}}$$

4 $${\text{YI}}=142.86\frac{b*}{{L}^{*}}$$

where $\Delta {L}^{*}$, $\Delta {a}^{*}$ and $\Delta {b}^{*}$ refer to the difference between L*, a* and b* values of the films and the white calibration plate (L*: 93.76; a*: − 1.05; b*: 0.74).

#### Mechanical properties

The samples were cut into strips of 2.5 cm × 7.5 cm and tested for their mechanical characteristics. TS and EB values were determined by a texture analyzer (TA-XT2i, Stable Micro Systems,UK) equipped with a 50 kg load cell and mini tensile grips A/MTG (Stable Micro Systems, UK). The film strips were stretched with a crosshead speed of 1 mm/s at room temperature (~ 23 ± 1 °C).

#### Fourier transform infrared spectroscopy (FTIR) analysis

The FTIR spectra of the samples (2 cm × 2 cm) were recorded in the wave number region between 650 and 4000 cm^−1^ by a FTIR spectrometer (Spectrum 100, Perkin Elmer, UK) (Gökkaya Erdem and Kaya 2022).

#### Thermal properties

The thermal characterization of the films was carried out using a differential scanning calorimeter (DSC 4000, Perkin Elmer, USA) at a nitrogen flow of 50 mL/min. 3–5 mg of film was hermetically sealed in an aluminum pan and scanned from 25 to 400 °C. The heating rate was 10 °C/min.

#### Film biodegradation in soil

To evaluate the film’s biodegradability, the method reported by Zhao et al. (2019) was used with minor revisions. Firstly, the film samples (2 cm × 2 cm) were dried at 50 °C in a vacuum oven for 24 h. Soil up to 40 mm high was filled into a plastic tray. Then, the dried films were buried in the soil to a depth of 10 ± 1 mm. The films were kept at room temperature for 12 days. Water (50 mL) was sprayed daily onto the soil to preserve the moisture. The degraded films were removed from the soil every 2 days, rinsed with water, and dried at 50 °C for 24 h. Dried films were weighed, and the weight loss of the films was calculated.

#### Light transmittance

The light transmittance of the films was measured by a spectrophotometer (UV-3600 Plus, Shimadzu, Japan). The films were scanned from 190 to 900 nm.

#### Water vapor permeability (WVP)

The American Society for Testing and Materials (ASTM) E96-00 method (ASTM 2000) was applied to analyze the WVP of the films. Cylindrical glass containers (4.5 cm diameter and 2.8 cm height) were filled with silica gel (20 g, 0% RH) with a headspace of 1 cm. The films were sealed on the containers by using epoxy glue (Pattex), and the initial weights of the containers were measured. Then, the film-sealed containers were put in a desiccator comprising distilled water (100% RH), and the desiccator was stored at 30 °C. The containers were weighed eight times over an 8-h period, and a graph was plotted showing the mass change as a function of time. The water vapor transmission rate (WVTR) was determined using the slope of the straight line (g/h) divided by the water permeation area (m^2^). The WVP of the films was determined using Eq. 5.

5 $$ {\text{Water }}\;{\text{vapor }}\;{\text{permeability }}\;\left( {{\text{WVP}}} \right) = \frac{{{\text{WVTR}} \times {\text{L}}}}{{{\Delta P}}} $$

where; WVP is the water vapor permeability (g mm m^−2^ h^−1^ kPa^−1^), WVTR is the water vapor transmission rate (g m^−2^ h^−1^), L is the film thickness (mm) and $\Delta P$ is the partial pressure difference (kPa) across the two sides of the film.

### Statistical analysis

The results are reported as the mean ± SD of triplicate observations. The differences between the testing parameters were evaluated statistically by independent samples *t*-test using SPSS software (SPSS Inc., Chicago, IL, USA, Version 25). The results were assessed at the *p* < 0.05 significance level. After the optimization procedure, the reliability of the predicted and experimental data were verified using One Sample *t*-test.

## Results and discussion

### Optimization of the film composition

The effect of independent factors and their interactions on the FWS, TS, and EB of films were defined by RSM using a central composite face-centered design (CCFD). Table 1 shows the produced CCFD and the observed experimental response values. Multiple regression analysis was used to statistically model the film production conditions. For FWS, the two factor interaction (2FI) model was the best fitting model for the experimental values. Moreover, the quadratic model was chosen for the TS, and EB as suggested by the software. The insignificant parameters and interaction terms were excluded from the models by using backward elimination. The final empirical models for FWS, TS and EB were specified in Eqs. (6), (7) and (8), respectively, in terms of coded factors:

6 $$FWS \left(\%\right)=45.94+3.74\times {X}_{1}+5.85\times {X}_{2}-2.85{\times X}_{1}\times {X}_{2}$$

7 $$TS=9.41-7.04\times {X}_{1}-4.15\times {X}_{2}+1.51\times {X}_{1}\times {X}_{2}+4.63\times {X}_{1}^{2}-2.92\times {X}_{2}^{2}$$

8 $$EB \left(\%\right)=37.71-2.97\times {X}_{1}+13.01\times {X}_{2}-6.12\times {X}_{1}\times {X}_{2}-25.45\times {X}_{1}^{2}$$

The developed models were statistically tested using ANOVA (Table 2). The results showed that the models are statistically significant with high *F*-values, low *p*-values, and the non-significant lack of fit tests. The coefficient of determination (R^2^) values were 0.9813, 0.9877 and 0.9627 for FWS, TS and EB, respectively, indicating that there was a strong correlation between the experimental and predicted values from the models. The two linear model terms X_1_ and X_2_ and their interactive effects (X_1_*X_2_) were significant (*p* < 0.05) for FWS and TS, showing that the hemicellulose/gelatin ratio and glycerol ratio affect FWS and TS significantly. The quadratic (X_1_^2^ and X_2_^2^) effects of variables were also significant (*p* < 0.05) for the TS of the films. On the other hand, X_2_, X_1_*X_2_ and X_1_^2^ were the significant (*p* < 0.05) model terms, while the hemicellulose/gelatin ratio (X_1_) was not significant (*p* > 0.05) for EB.

**Table 2:** Statistical parameters of the models

Source	Film water solubility		Tensile strength		Elongation at break	
	F-value	*p*-value	F-value	*p*-value	F-value	*p*-value
Model	157.38	< 0.0001^a^	112.22	< 0.0001^a^	51.59	< 0.0001^a^
X_1_	123.04	< 0.0001^a^	352.30	< 0.0001^a^	3.29	0.1071^b^
X_2_	301.51	< 0.0001^a^	122.60	< 0.0001^a^	63.26	< 0.0001^a^
X_1_*X_2_	47.59	0.0166^a^	10.89	0.0131^a^	9.35	0.0156^a^
X_1_^2^			70.22	< 0.0001^a^	130.45	< 0.0001^a^
X_2_^2^			27.86	0.0012^a^		
Lack of fit	3.87	0.1072^b^	3.50	0.1287^b^	4.33	0.0925^b^

The R^2^ values for the models were 0.9813, 0.9877 and 0.9627 for FWS, TS and EB, respectively

^a^Significant at *p* < 0.05

^b^Not significant at *p* > 0.05

**Fig. 1:**
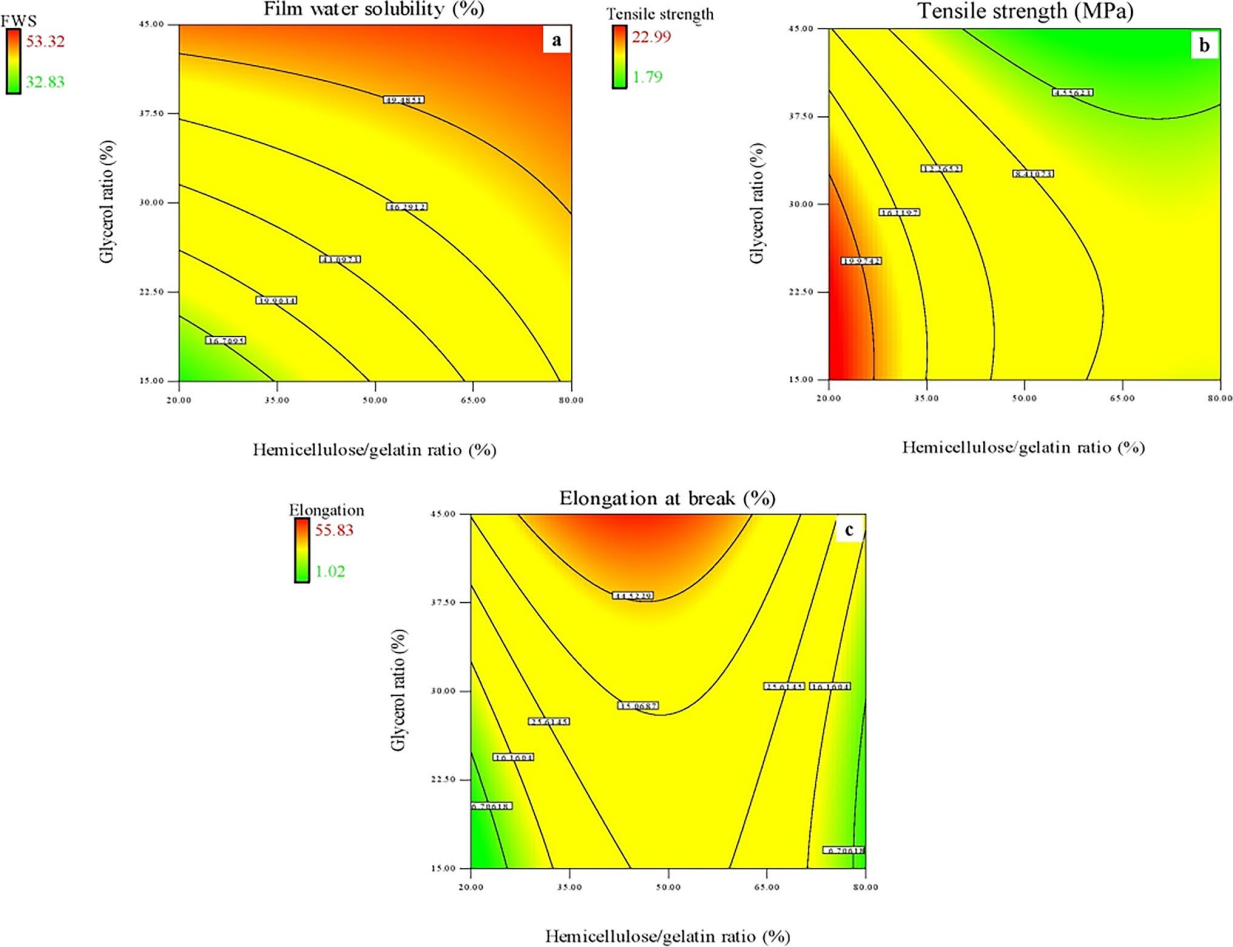
Contour plots presenting the interactive effects of the independent factors on **a** film water solubility (FWS), **b** tensile strength (TS) and **c** elongation at break (EB)

The FWS indicates the resistance of films against water when applied (Cerqueira et al. 2012). FWS of the hemicellulose-gelatin films varied from 32.83 to 53.32% (Table 1). These results were compatible with the results of Lee et al. (2004). According to their results, the FWS values of the gellan/gelatin films changed from 30 to 52%. Kocabaş et al. (2021) pointed out that the FWS values of nanocellulose-reinforced hemicellulose films ranged from 50.80% to 61.49%. Figure 1 represents the response surface contour plots of the generated models, which were constructed to show the effects of independent factors and their interactions on FWS, TS, and EB. It is seen that FWS increases with the increasing hemicellulose/gelatin ratio (Fig. 1a). The increase in FWS with the increasing amount of hemicellulose could be explained by the hydrophilic structure of hemicellulose. Glycerol also enhanced the FWS of the hemicellulose-gelatin films. Similar results were observed by Nor et al. (2017), who determined the effect of glycerol concentration on the water solubility of chicken skin gelatin films. They explained this behavior by the hydrophilic and highly soluble nature of glycerol. Similar observations were also reported for cellulose nanofiber-reinforced hemicellulose/chitosan films by (Xu et al. 2019), chitosan, and galactomannan films (Cerqueira et al. 2012).

TS and EB values are important and widely measured parameters used to define the performance of packaging materials (Kocabaş et al. 2021). TS is the capability of a material to resist tensile stress until it breaks, and EB can be defined as the percentage increase in the length of a material before it breaks under tension (Cerqueira et al. 2012). The hemicellulose-gelatin films produced in this study exhibited TS of 1.79 to 22.99 MPa and EB of 1.02–55.83% (Table 1). Ahmad et al. (2021) stated the TS and EB values of arabinoxylan-gelatin films as 3.62–4.12 MPa and 83.5–97.4%, respectively. While the hemicellulose/gelatin ratio significantly affected the TS of the films (*p* < 0.05), its effect on the EB was insignificant (*p* > 0.05). A decreasing trend in the TS of the samples was observed with the increasing hemicellulose/gelatin ratio (Fig. 1b). The decreasing TS could be described by the decreased interaction between the molecular chains of the film. Also, in a study performed by Ahmad et al. (2021), it is stated that the addition of gelatin to arabinoxylan film increased the TS of the samples. Adding plasticizer (glycerol) increased the EB while decreasing the TS of the films. This situation may be due to the fact that the plasticizer decreased the intermolecular forces between hemicellulose and gelatin, softened the stiffness of the film structure, and increased the chain mobility. Decreasing TS and increasing EB values with the presence and increasing plasticizer concentration have been stated in other studies for different types of films (Cerqueira et al. 2012; Xu et al. 2019).

The composition of the film forming solution was optimized to obtain lower FWS and higher TS and EB values. The optimal conditions were selected as hemicellulose/gelatin ratio of 35.93% and glycerol ratio of 18.02%, and the FWS, TS and EB values of the samples were predicted as 38.45%, 15.75 MPa and 21.76%, respectively. To verify the results predicted by the model, three films were prepared under the optimum conditions. The experimental FWS, TS and EB values were 39.21%, 16.64 MPa and 20.59%, respectively. In addition, One-Sample *t*-Test presented that the experimental values were not significantly different from the values suggested by the program (*p* > 0.05) which indicates the reliability of the predicted conditions by RSM.

### Moisture content, thickness and water solubility of the films

The moisture content of the hemicellulose-gelatin film was 6.34% (wb). No significant difference (*p* > 0.05) was detected between the moisture content values of the films (Table 3), that means that the hemicellulose incorporation did not affect the film moisture content.

**Table 3:** Gelatin and hemicellulose-gelatin films properties

Sample	Gelatin film	Hemicellulose-gelatin film
Moisture content (%, wb)	6.31 ± 0.17^a^	6.34 ± 0.08^a^
Thickness (mm)	0.092 ± 0.012^a^	0.089 ± 0.010^a^
FWS (%)	49.57 ± 1.01^a^	39.21 ± 1.76^b^
Tensile strength (MPa)	20.41 ± 0.78^a^	16.64 ± 0.92^b^
Elongation at break (%)	4.74 ± 0.34^b^	20.59 ± 0.86^a^
WVP (g mm/m^2^ h kPa)	0.160 ± 0.004^b^	0.247 ± 0.003^a^
*Color parameters*		
L*	72.473 ± 0.16^a^	47.098 ± 0.62^b^
a*	− 1.173 ± 0.02^b^	18.973 ± 0.65^a^
b*	2.843 ± 0.02^b^	30.623 ± 1.26^a^
ΔE*	72.538 ± 0.16^a^	59.309 ± 0.36^b^
WI	72.301 ± 0.16^a^	35.993 ± 1.31^b^
YI	5.603 ± 0.04^b^	92.937 ± 5.04^a^

Different lowercase letters (a, b) represent statistical difference between the two films using the independent samples *t*-test (*p* < 0.05)

The film thickness and water solubility are important parameters which have an impact on food packaging. According to Mendes et al. (2017), the film thickness ensures the uniformity, precision, and accuracy of the film production process. There was no significant difference between the thickness values of the films (*p* > 0.05) (Table 3), this could be explained by the uniformity of the produced films. The incorporation of hemicellulose didn’t affect the film thickness, indicating that the process of water removal was uniform for all samples.

The water solubility of the films was 49.57% and 39.21%, respectively, for gelatin and hemicellulose-gelatin film, with a significant difference (*p* < 0.05). As is known, gelatin is highly hygroscopic and therefore, gelatin films have poor water resistance (Weng and Zheng 2015). In this study, incorporation of hemicellulose into gelatin decreased the film solubility. Hemicellulose contains a large amount of free hydroxyl groups and these groups generate intermolecular and intramolecular hydrogen bonds (Balli et al. 2019). The lower solubility of gelatin film incorporated with hemicellulose might be explained by the strong network between gelatin and hemicellulose because of the ability of hemicellulose to form hydrogen bonds (Kocabaş et al. 2021). The decreasing film solubility improves the film quality and widens its applications in food packaging.

### Mechanical properties

The mechanical properties of packaging materials provide important information about the ability of the material to maintain its integrity under the influence of various stresses occurring during the processing, transportation, and storage of packaged foods (Bastarrachea et al. 2011). The hemicellulose-gelatin film had a TS of 16.64 MPa with an EB of 20.59%, while these values were 20.41 MPa and 4.74%, respectively, for the gelatin film. The hemicellulose incorporation into the gelatin film significantly decreased TS and increased EB (*p* < 0.05) (Table 3). That means the incorporation of hemicellulose improved the flexibility of the gelatin film, which made its application in food packaging easier. Because, flexible packaging materials have many advantages in the food industry, resulting from their low weight, formability, multilayer complexity, and cost (Bamps et al. 2023). Ahmad et al. (2021) reported the TS and EB values of arabinoxylan-gelatin films in the range of 3.62–4.12 MPa and 83.5–97.4%, respectively.

### Water vapor permeability

The choice of the packaging materials depends on some criteria, such as the hydrophobicity, which is the most essential one because of the product performance as well as its sensitivity to water. The films need to have low WVP as well as reduce moisture transfer between the food and the outer atmosphere. As shown in Table 3, WVP increased significantly (from 0.164 to 0.247 g mm/m^2^ h kPa) with the hemicellulose incorporation into gelatin (*p* < 0.05). The high WVP of hemicellulose-gelatin film can be described by the hydrophilic polymers present in the film formulation. The hydrophilic nature of polymers makes them very accessible to moisture, resulting in poor water vapor barrier propertie,s as reported by several authors. The result found in this study is consistent with the study of Loo and Sarbon (2020), who stated that adding tapioca starch to the chicken skin gelatin based films significantly increased the WVP of the films.

### Color properties

Color is one of the major attributes that affect the consumer’s perception of the quality of food packaging. Table 3 shows the color variability for gelatin and hemicellulose-gelatin films. Regarding a* and b* values, a significant difference (*p* < 0.05) was obtained between the two films. Concerning the b* values, their increase resulted in a similar increase in the yellowness index (YI). The obtained results mean that the incorporation of hemicellulose increases red and yellow intensity, while a different trend was observed for the lightness values (L*). This decrease in lightness was approved by a similar decrease in whiteness index (WI). Thus, it was expected because of the hemicellulose powder color. Regarding the changes in all the color values, we observed a huge variation between the two films, indicating that the addition of hemicellulose extracted from pistachio shells causes changes in the visual appearance of the films. The obtained results are similar to the findings of Luo et al. (2022), who noticed an improvement in the color parameters with the addition of some components to the colorless gelatin film.

### Light transmittance

Light exposure is known for its potential to catalyze some reactions that cause unfavorable effects on foods (oxidation of fats and oils, formation of off-flavors, discoloration, and decrease in vitamins (A, B, C) rates). The most obvious effects are generally detected in the lower wavelengths of the visible region and the UV region. The light transmittance of the films gradually increased from the UV region (190–400 nm) to the visible region (400–900 nm) (supplementary Fig. 1). According to the obtained results, the transmittance at 400 nm of gelatin film is higher than that of hemicellulose-gelatin film by 4.59 times (84.73% and 18.47%, respectively). In the visible region, the gelatin film reached maximum transmittance at 621 nm, while the hemicellulose-gelatin film reached it at 807 nm, providing lower light transmittance.

The incorporation of hemicellulose into the gelatin film decreases significantly the optical transparencies. As stated by Chadni et al. (2020), hemicellulose-based films, obtained by alkaline extraction conditions, usually show low transmittance in the visible region. In accordance with these results, the addition of hemicellulose to the gelatin film indicated high light barrier properties in the UV and visible regions. Considering the damage caused by light exposure to food products (shelf-life, quality, nutritional, and organoleptic properties), hemicellulose-gelatin film could be a suitable alternative for food packaging.

### Fourier transform infrared spectroscopy (FTIR)

**Fig. 2:**
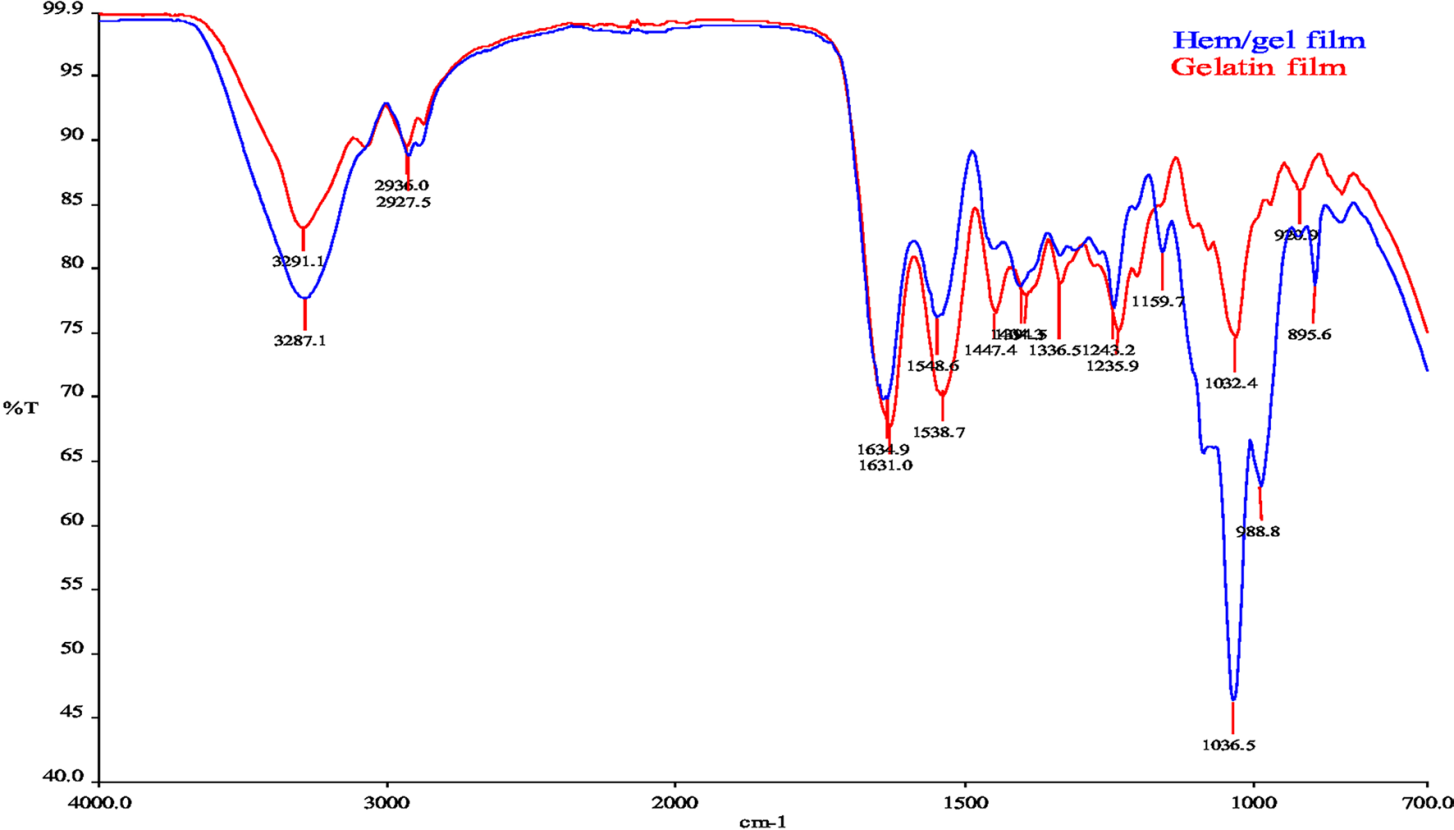
FT-IR spectrums of gelatin and hemicellulose-gelatin film

The FTIR spectrum of films was recorded between 700 and 4000 cm^−1^. The IR spectra was released with the aim of understanding the structural changes that occur in films with the incorporation of hemicellulose (Fig. 2). A broad absorption band around 3300 cm^−1^, could be assigned to O–H stretch vibration (Yang et al. 2006). As illustrated by Pandey (1999), the peak realized around 2900 cm^−1^ is related to stretching of the C–H aliphatic bond and the peak at ~ 1630 cm^−1^ corresponds to O–H bending vibrations of absorbed water. Starting in this region, the incorporation of hemicellulose amplified the intensity rate. The several absorption bands present between 1400 and 1500 cm^−1^ could be associated with C = C bonds in aromatic rings and the bending vibrations of C–H (Yang et al. 2006). The peaks between 1200 and 1250 cm^−1^ might be due to the stretching vibrations of the primary and secondary alcohols C–O. This stretching vibration could be a result of the interaction with the plasticizer (glycerol). As known, glycerol decreases the intermolecular forces between polymeric chains, allowing a greater absorption of water molecules (Carneiro-da-Cunha et al. 2009). At 1036.5 cm^−1^ the hemicellulose-gelatin film intensity declined sharply. This sharp band could be explained by the presence of xylans found at 1034–1040 cm^–1^ (Egüés et al. 2013). The FTIR spectra of hemicellulose-gelatin film showed bands at around 895 cm^−1^, which could be assigned to the stretching of α-D-xylose linkages and β-D-galactose in hemicellulose, as reported by Mendes et al. (2017). In addition, they attribute the region between 1200 and 800 cm^−1^ to the “fingerprint” region of hemicellulose.

### Thermal properties of films

**Fig. 3:**
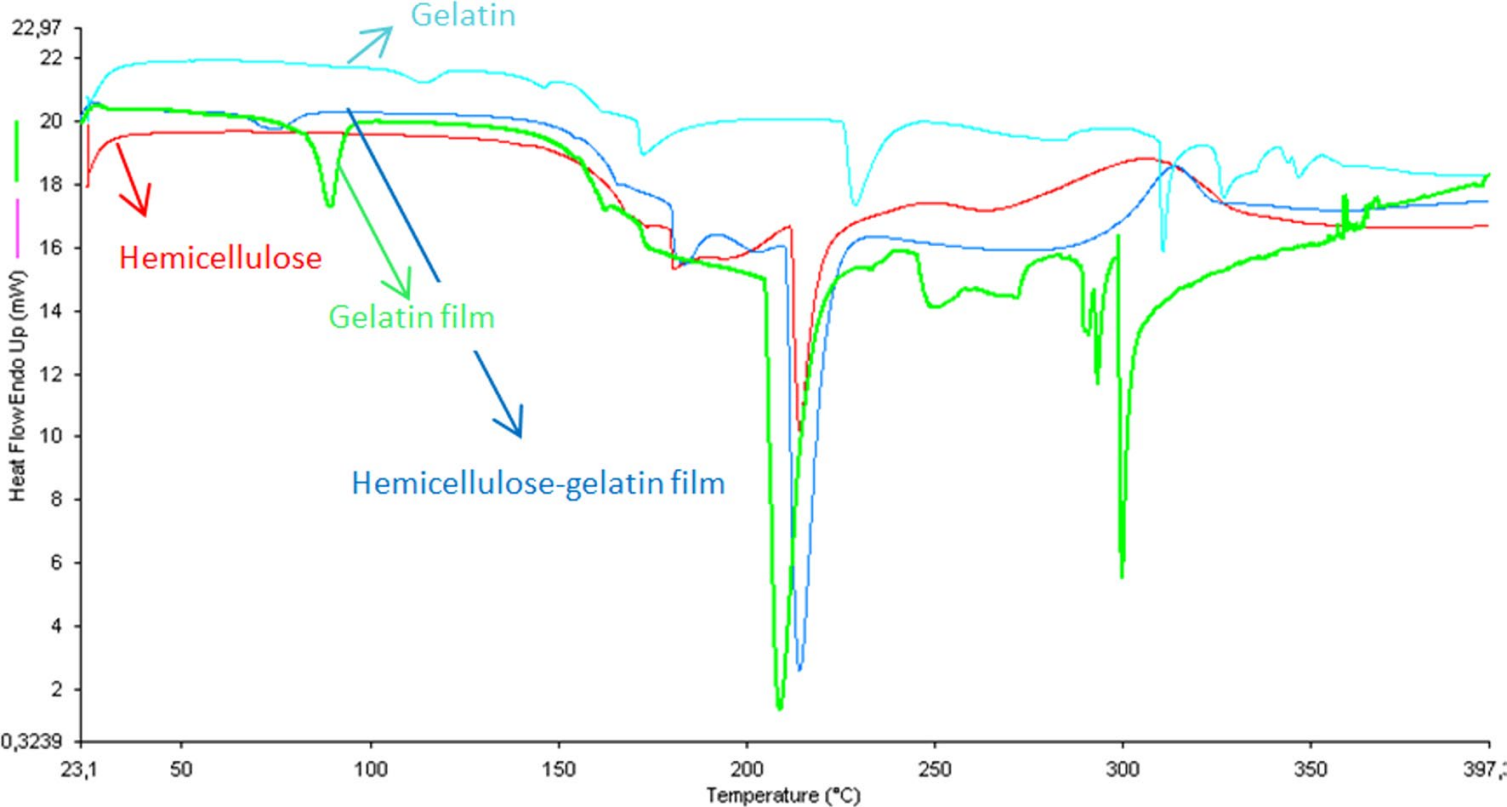
DSC thermograms of hemicellulose, gelatin, gelatin film and hemicellulose-gelatin film

The first heating DSC thermograms of hemicellulose, gelatin, gelatin film, and hemicellulose-gelatin film are given in Fig. 3. The glass transition was not observed in the measurement range of the xylan-based hemicellulose thermogram. Thermogram was showing a sharp endothermic signal with a peak position at 214 °C, while the two wide exothermic peaks at 247 °C and 307 °C followed the endothermic signals. Similar exothermic signals in the DSC thermogram of xylan-based hemicellulose have been explained as polymer decomposition and degradation reactions (Ünlü et al. 2012). The first degradation peak is due to the decomposition of the side units, such as acetyl groups, while the second one can be due to the decomposition of the xylan backbone. However, the first endothermic signal can be attributed to the presence of lignin and/or phenolic compounds in the extracted xylan-based hemicellulose samples (Göksu 2005). The hemicellulose-film thermogram showed a slightly different pattern compared with that of the hemicellulose thermogram and the major endothermic peak at 214 °C was almost the same. An extra small endothermic peak at 74.73 °C was observed in hemicellulose film due to the melting of gelatin. The first exothermic depolymerization peak disappeared in the hemicellulose-gelatin film, while the second one shifted to a higher temperature, from 307 to 313 °C. Hence, it can be concluded that hemicellulose-gelatin film was more thermally stable than hemicellulose. DSC thermograms of commercial gelatin showed that it undergoes many transition during heating from 30 to 400 °C. The glass transition was observed at around 83 °C and the following endothermic signal at around 114 °C can be attributed to the thermal unfolding of gelatin. Commercial gelatin also shows melting isotherm and isomerization at around 173 °C and 228 °C, respectively. Moreover, some other endothermic transitions at higher temperatures (above 300 °C) can be attributed to the decomposition of gelatin. A similar pattern was earlier reported for bovine gelatin by Rahman et al. (2010). The glass transition peak disappeared in the gelatin film, which is quite different from the gelatin thermogram. The melting and isomerization peaks merged into a single peak at approximately 209 °C. The thermal unfolding and decomposition peaks moved to lower temperatures, around 90 °C and 290 °C, respectively. This behavior can be associated with the plasticizing effect of glycerol. Because plasticized gelatin behaves as dispersed molten plastic. Hence, plasticization of gelatin may result in a lowering glass transition temperature and unfolding. When hemicellulose-gelatin film was compared with gelatin film, it was observed that gelatin degradation peaks disappeared. It might be because of the good interaction of hemicellulose with gelatin.

### Film biodegradation

The produced films were buried in soil for 12 days to test their biodegradability. On the second day, the hemicellulose-gelatin film had higher weight loss than the gelatin film (35 and 15%, respectively) (supplementary Fig. 2). The weight loss increased twice with the incorporation of hemicellulose into the gelatin film. After 12 days, the biodegradability of the hemicellulose-gelatin film reached to 49.62%, while the gelatin film was degraded by about 23.72%. These findings show that the addition of hemicellulose to gelatin significantly enhanced the biodegradability of the film. Although there is no study in the literature on the biodegradation of hemicellulose-gelatin film, it has been reported that both hemicellulose and gelatin films have high biodegradability as a result of their hydrophilic nature (Kocabaş et al. 2021). Considering the good biodegradability of prepared hemicellulose-gelatin film, it can replace the traditional petroleum-based packaging materials in food packaging applications to reduce environmental problems.

## Conclusion

This study concluded that the hemicellulose extracted from pistachio shells was added to a gelatin-glycerol film to improve its properties. Different hemicellulose/gelatin and glycerol ratios were studied to obtain a good combination of physical properties for potential use as a biodegradable food packaging material. The optimum conditions were defined as 35.93% of the hemicellulose/gelatin ratio and 18.02% of the glycerol ratio to obtain lower film water solubility, higher tensile strength, and elongation at break values. The incorporation of hemicellulose in gelatin decreased the water solubility and the tensile strength while improving the film flexibility. The incorporation of hemicellulose into gelatin film caused changes in the visual appearance of the films and enhanced the film water vapor permeability, while no significant difference was noticed in moisture content or thickness values for both films. Regarding the biodegradation properties, the hemicellulose-gelatin film showed much more rapid degradation than the gelatin film. Thus, the obtained results suggested that the hemicellulose-gelatin films have satisfying properties compatible with food-related packaging applications. Furthermore, the hemicellulose was extracted from pistachio shells, which extend the valorization of that agricultural waste. The use of these materials permits edible, sustainable, biodegradable, non-toxic, low-cost, renewable, and eco-friendly packaging. Further studies should be conducted to evaluate the application of gelatin-based films incorporated with pistachio shell hemicellulose in the packaging of various food products.

## Supplementary Information

Below is the link to the electronic supplementary material.

Supplementary file1 (DOCX 59 KB)

## Data Availability

Data will be made available on reasonable request.
