# Supplementary material for: Development and characterization of gelatin-based biodegradable films incorporated with pistachio shell hemicellulose
Source: J Food Sci Technol. 2024 Mar 17;61(10):1919–29. doi: 10.1007/s13197-024-05968-4 (PMC11401810; doi:10.1007/s13197-024-05968-4)
Supplement: Supplementary file 1 — Supplementary file1 (DOCX 59 KB) [file 13197_2024_5968_MOESM1_ESM.docx]

**Supplementary Material**

**Development and characterization of gelatin-based biodegradable films incorporated with pistachio shell hemicellulose**


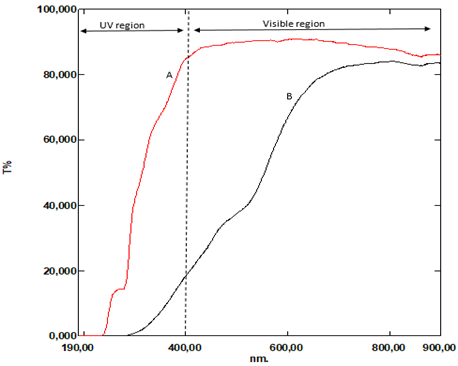


**Fig. S1** Transmittance (%) curves of gelatin film (A) and hemicellulose-gelatin film (B).

**Fig. S2** Biodegradation of gelatin and hemicellulose-gelatin film
